# Effects of the Change in Working Status on the Health of Older People in Japan

**DOI:** 10.1371/journal.pone.0144069

**Published:** 2015-12-03

**Authors:** Ushio Minami, Mariko Nishi, Taro Fukaya, Masami Hasebe, Kumiko Nonaka, Takashi Koike, Hiroyuki Suzuki, Yoh Murayama, Hayato Uchida, Yoshinori Fujiwara

**Affiliations:** 1 Research Team for Social Participation and Community Health, Tokyo Metropolitan Institute of Gerontology, Tokyo, Japan; 2 Graduate School of Human Science and Environment, University of Hyogo, Hyogo, Japan; 3 Department of Translational Research Promotion, Tokyo Metropolitan Institute of Gerontology, Tokyo, Japan; 4 Faculty of Human Welfare, Seigakuin University, Saitama, Japan; 5 College of Humanities and Sciences, Nihon University, Tokyo, Japan; Hamamatsu University School of Medicine, JAPAN

## Abstract

**Background:**

Working at old ages is regarded as a good way to keep one’s health according to the idea of productive aging. However, there is not enough evidence yet whether retirement is good or bad, or the kind of effects it has on the health of older adults aged 65 and over. We examined it by using a recent data of Wako city, a suburb area near Tokyo in Japan.

**Methods:**

One thousand seven hundred sixty-eight participants answered to 3 waves of survey questionnaires: 2008, 2010, and 2012, successively. We considered 3 indicators of health; self-rated health, mental health (GDS15) and HLFC (Higher-Level Functional Capacity: TMIG-IC). In cross-sectional analysis, we compared these 3 indicators by three groups: full-time worker, part-time worker, and non-worker. In longitudinal analysis, we compared these three indicators by two groups: subjects who successively worked in 2008, 2010, 2012, and subjects who worked in 2008 but retired before 2010. We used one-way and two way repeated measures ANCOVA for these analyses, respectively.

**Results:**

It was significantly clear that retirement worsened both mental health and HLFC in people aged 65 years and over; especially, mental health worsened rapidly and HLFC gradually. However, these indicators didn’t worsen in subjects who changed from full-time jobs to part-time jobs. Quitting from part-time jobs deteriorated mental health gradually and HLFC moderately compared to full-time jobs.

**Conclusion:**

The results support the activity theory that older adults who quit from full-time jobs deteriorated both mental health and HLFC, though at different speeds. If they make a transit to part-time jobs, the deterioration would be moderate. It shows that working is an effective way of social participation for older people aged 65 years and over in Japan.

## Introduction

According to OECD (Organization for Economic Co-operation and Development) statistics, Japan is the seventh in the 35 countries of OECD where the working rate of the elderly people is high. [1]. As the proportion of elderly over 65 years old is estimated to increase rapidly and, on the contrary, the number of labours decrease, the Japanese government is promoting older people to engage into work [2]. “The Law concerning Stabilization of Employment of Older Persons” was amended in April 2013; therefore, those who hope to work until 65 years of age are protected to do so.

In Japan, the employee used to have a lifelong commitment to their first employment until retirement after 1950s. When the seniority system was predominant, there were opinions that older people should retire as early as possible not to disturb younger people [3]. There are many studies indicating that retirement has either a good effect or no relation at least on one’s health [4],[5],[6],[7],[8],[9] which supports disengagement theory [10]. On the other hand, there are also many researches suggesting that retirement worsens one’s health [11],[12],[13],[14],[15],[16], which supports activity theory [17].

Disengagement theory and activity theory are the diametrically opposite notions about the relationship to the society for older people which have been disputed for a long time in gerotological study. According to the disengagement theory, it is natural for older people to withdraw from society gradually as they age. Because they become short of stamina, the mental and physical health improves by leaving hard work. On the other hand, according to the activity theory, maintaining relations with the society leads to successful aging. By this notion, withdraw from the society by retirement deteriorates one’s health.

The subjects of the former researches in Japan [8],[9] were restricted as full-time regular workers and the subjects of the latter [16] were under 65 years old. There is no Japanese research focusing on the people aged 65 and older except for the one indicating that work may protect a decline in BADL only in men, by using an 8-year longitudinal survey [18]; however, the data used was from the year 1990s. In Japan, we have experienced rapid ups and downs of the economy in the last twenty years and this, in turn, has violently changed the employment environment, generating either many willing and unwilling early retirees or other flexible and diversified types of employment such as non-regular workers [19]. People’s attitude toward retirement may drastically change, but it has not been verified in the last these fifteen years. Moreover, there are few researches focusing on the people aged 65 years and over who have retired in Japan.

Precisely, the current working status of the people over 65 years and older is not clear enough. What kind of people are working or how they are working in Japan? The Silver Human Resource Center was the largest independent employment support system in Japan to share works with older people, collaborating with many public institutions or local authorities [20]. However, the works the members share are restricted to low wage and light work within 20 hours a week and the number of its members is about 730 thousands nationwide which is only about 2% of elderly people in Japan [21]. The statistics shows that about 19.5% of the elderly people are working [1], but it is not clear yet about the rest of them.

Moreover, due to the employment environmental change in the last twenty years, many alternatives have occurred in older workers, such as going to Hello-Work (public employment security office with no age restriction) [22], using commercial temporary staff services, starting a new business by themselves, or engaging in Nonprofit Organizations. Recently, they can also get allowances to participate in other social activities as volunteers (to get vouchers, regional money, or paid vacation). There, they can work not as only full-time workers but also part-time or telecommuting workers [23]. Especially in older job seekers, there are few job offers of regular employee, but mainly part-time employee in opposition to their intentions [22]. It is also not clear the difference of the effects by these types of employment yet.

Also, we have to recognize the different situations between those working under 65 years old and over 65 years old. According to the Annual white paper on the Aging Society 2014, 71.0% of the people over 60 years old in Japan don’t feel any trouble at their life economic situations [24]. They tend to start their second lives after ensuring enough assets, monthly steady pensions and social securities. At their first retirement, people may be anxious about their financial situation, but it is more important for them how to spend their rest of life at the retirement after 65 years old, because the financial situation cannot be changed easily by any means after 65 years old. Moreover, the work they can do is limited by the physical conditions even though they have enough experiences, and they are less ambitious about the economical attainments than younger generations. Older people prefer to emphasize their contribution to society at working. It is a kind of health promotion or participation to the regional community for improving their well-being [25]. Social participation at older age is regarded to have good effects for one’s health as a preventive measure from isolation. It is expected even to reduce medical expenses. This study aims to clarify the effect of working as a way of social participation and change in the working status on the health of the people over 65 years old using the data that reflects the change of employment environment these days.

## Materials and Methods

The occupations the older people engage in are very different between in urban and rural areas. Agriculture, forestry, and fishery are main fields of working at older age in rural areas but not in urban areas [18]. Wako city is located at the South-East part of Saitama prefecture which has been developed as a bed town within the range of 15–20 kilometers directly connected to Tokyo metropolitan area by three railways. Over 50% of workers or students living in Wako city commute to Tokyo. Agricultural lands are about 11.1% of the city area [26].

In 2008, the population of this city was 74,879 and that of those over 65 years old was 10,003. The study sample were 4,169 (41.7% of population) which consisted of two mail surveys (Wave 1-A and Wave 1-B), excluding the elderly living in institutional care facilities and registered as Care-Level 2 whose criteria was partially dependent in basic activities of daily livings such as toileting or feeding, and more severe from all the elderly 65 years and older living in the city. Wave 1-A complied of representative samples of 2,528 elderly over 65 years old who were randomly selected residents of Wako-City on July 1st in 2008. Wave 1-B complied of all residents registered as living alone on October 1st in 2008, exclusive of Wave1-A subjects (n = 1,641). We collected completed replies from 1,773 subjects (70.1%) of Wave 1-A and 1,141 subjects of Wave 1-B (69.5%). We followed up these subjects with identified ID number. This survey questionnaires were conducted by mail at three different times 2008 (Wave 1), 2010 (Wave 2), and 2012 (Wave 3) in this city. We analyzed the answers of 1,768 people with continuous valid responses at these 3 times.

### Ethical considerations

This research had been all approved by the Ethics Committee (IRB: Institutional Review Board) of the Tokyo Metropolitan Institute of Gerontology. Participants were explicitly informed that the data produced in the survey would be confidential, would not affect their treatment, and would be used only for academic research purpose. All participants gave their informed written consents to each survey by themselves. We didn’t accept the consent by any surrogate.

### Measurements

We used the following four variables at 3 waves in this study.

1. Working Status
  Respondents were asked about their working status at each wave from the three options: “Working more than 35 hours per week”, “Working less than 35 hours per week”, and “Not working”. We regarded “Working more than 35 hours per week” as a full-time worker (F), “Working less than 35 hours per week” as a part-time worker (P), and “Not working” as a non-worker (N).
2. Self-rated health
  Self-rated health is a subjective opinion about one’s general health status which is asked at the beginning of the questionnaire, such as “how do you feel about your health status?” from four options of “very good”, “good”, “fair”, and “poor”. We categorized the options: “very good” and “good” as 1 and “fair” and “poor” as 0 with regard to the data analysis.
3. Mental Health: GDS15
  We use the Japanese version of Geriatric Depression Scale 15 (GDS15) to measure the respondents’ mental health. GDS15 is a screening tool for depression of older people [27]. It consists of 15 items with self-reported measures about feelings in daily life. The answers were labeled as ‘yes’ (1) or ‘no’ (0). Positive items are reverse coded (e.g., ‘Are you satisfied with your life?’ ‘Do you feel happy most of the time?’). Total scores range from 0 to 15 with higher scores indicating more depressive symptoms.
4. Higher-Level Functional Capacity (HLFC): TMIG-IC
  Tokyo Metropolitan Institute of Gerontology Index of Competence (TMIG-IC) is a widely used standard scale to measure HLFC in Japan and its validity has been tested repeatedly [28]. It evaluates 13 tasks about instrumental activities of daily living (5 items), intellectual activities (4 items), and social roles (4items). Each task was assessed with a "yes" or "no." Each "yes" counted as one point, for a maximum of 13 points. Higher scores reflected a higher functional level of higher-level competence.

### Procedure

At first, we compared self-rated health, mental health and HLFC by working status; full-time worker (n = 220), part-time worker (n = 273), and non-worker (n = 1275) at Wave1. Secondly, to understand the effect of the change of working status, full-time worker at Wave1 was set as the control variable, and we compared the following three groups: (F,F,F) which shows 55 people who continued to work as full-time workers at Wave2 and Wave3; (F,P,P) which shows 13 people who changed their status to part-time workers at Wave2 and Wave3; and (F,N,N) which shows 20 people who were unemployed at Wave2 and Wave3. Finally, we compared the two groups of (F,N,N) and (P,N,N) which shows 41 people who were part-time workers at Wave1 but become unemployed at Wave2 and Wave3, in order to clarify the difference of the effects of retirement from full-time job and part-time job. In our longitudinal analysis, we define the difference between Wave 1 and Wave 2 as a short time effect and the difference between Wave 1 and Wave 3 as a long-term effect.

### Statistical Analysis

In this study, we used one-way and two-way repeated measures ANCOVA (Analysis of Covariance) to distinguish the effects of each group. First, we compared the health related scores by working status as a cross-sectional analysis using one way ANCOVA. Sex, age, years of schooling, annual couple income, occupation (self-employed or not) were set as covariates. Secondly, to clearly differentiate the effects of the change in working status and time-effect, we compared three groups of (F,F,F)(F,N,N)(F,P,P) and two groups of (F,N,N)(P,N,N) by two-way repeated measures ANCOVA as longitudinal analyses using same covariates as a cross-sectional analysis. We analyzed all data with IBM SPSS statistics for Windows version 20.0 (IBM Corp., Armonk, NY, USA).

## Results

### Baseline Characteristics

Table 1 shows the baseline characteristics of the participants at Wave1 by working status. Full-time and Part-time workers were relatively younger than non-workers (p<0.001 by F test) and more males (p<0.001 by Chi-squared test). Part-time workers had higher educational attainments (p<0.001 by Chi-squared test). Annual couple income of full-time workers were higher than part-time workers or non-workers (p<0.001 by Chi-squared test). Percentage of self-employment was higher in full-time workers (p<0.001 by Chi-squared test).

**Table 1:** Baseline characteristics of the participants at Wave1.

		Total	Full-time	Part-time	None	p
		N = 1768	N = 220	N = 273	N = 1275	
Age	Mean(SD)	73.4(7.0)	70.2(4.8)	71.1(5.1)	74.3(7.3)	<0.001†
Sex	Male(%)	42.2	68.6	52.6	36.1	<0.001‡
Educational attainment	college degree or more (%)	25.6	26.2	30.7	24.6	<0.001‡
Annual couple income	≤2.99 million JPY(%)	64.5	38.8	55.9	70.5	<0.001‡
	3–4.99 million JPY	24.0	30.8	25.9	22.6	
	5–9.99 million JPY	8.3	20.3	12.1	5.5	
	≥10 million JPY	3.2	10.1	6.2	1.5	
Occupation	Self-employed(%)	16.2	41.2	22.7	10.5	<0.001‡

† by F test

‡ by Chi-squared tests

### Cross-sectional analysis

**Fig 1:**
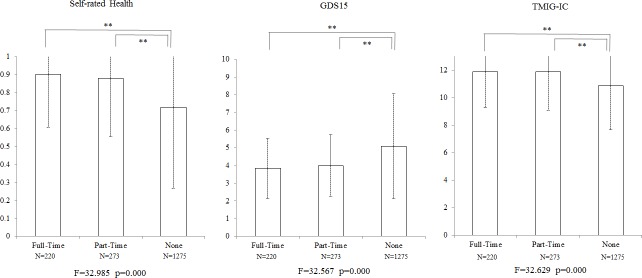
Cross-sectional analysis of working status at Wave1 by one-way ANCOVA with 5 covariates of age, sex, years of schooling, annual couple income, occupation (self-employed or not). ** p<0.01 by F tests.

Fig 1 shows the result of cross-sectional analysis at Wave1. Full-time and part-time workers were almost at a same level but significantly better than non-workers at self-rated health, GDS15, and TMIG-IC by one way ANCOVA (p<0.001) with the 5 covariates previously mentioned.

### Longitudinal analysis

**Fig 2:**
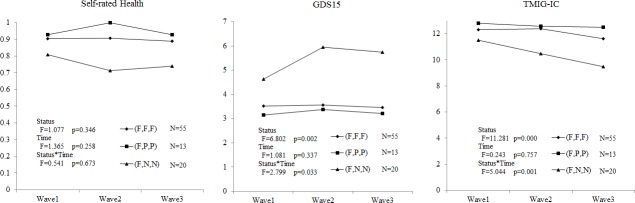
Longitudinal analysis of 3 groups by repeated measures ANCOVA. (F,F,F) shows the subjects who continued from Wave 1 to Wave 3 as full-time workers. (F,P,P) shows the subjects who changed from full-time work to part-time work between Waves 1 and 2. (F,N,N) shows the subjects who quitted their full-time work between Waves 1 and 2. Status: main effect of working status, Time: main effect of time series, Status*Time: interaction effects of working status and time series.

**Fig 3:**
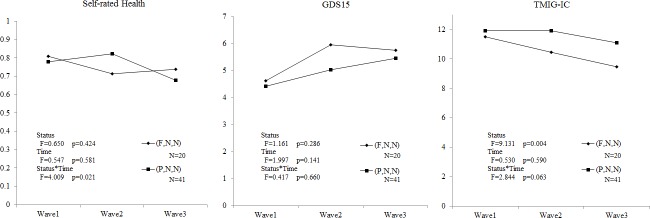
Longitudinal analysis of 2 groups by repeated measures ANCOVA. (F,N,N) shows the subjects who quitted their full-time work between Waves 1 and 2. (P,N,N) shows the subjects who quitted their part-time work between Waves 1 and 2.

Fig 2 shows statistically significant differences both in main effect (working status) and in interaction effect (working status by time) of GDS15 (p = 0.002, 0.033) and TMIG-IC (p<0.001, 0.001) between (F,F,F), (F,P,P) and (F,N,N). Fig 3 also shoes differences between (F,N,N) and (P,N,N) in the main effect (working status) of TMIG-IC (p = 0.004) and interaction effect (working status and time) of self-rated health (p = 0.021) (Table A and Table B in S1 File).

Table 2 is the result of multiple comparisons with Bonferroni corrections. We found significantly different types of appearance between mental health (GDS15) and HLFC (TMIG-IC) in a short-term and long-term between Wave1 and Wave2 (p = 0.002, <0.001), and between Wave2 and Wave3 (p = 0.699, 0.012) of (F,N,N). The effects on mental health emerged in a short-term, though the effects on HLFC gradually continued in a long-term.

**Table 2:** Results of multiple comparisons by two-way repeated measures ANCOVA.

(F,F,F)(F,P,P)(F,N,N)							
Self-rated health							
Status	W1	W2	W3	Time	(F,F,F)	(F,P,P)	(F,N,N)
(F,F,F)_(F,P,P)	1.000	1.000	1.000	W2_W1	1.000	0.487	0.551
(F,P,P)_(F,N,N)	0.656	0.307	1.000	W3_W1	0.719	1.000	0.552
(F,F,F)_(F,N,N)	0.528	0.612	1.000	W3_W2	0.599	0.317	0.087
GDS							
Status	W1	W2	W3	Time	(F,F,F)	(F,P,P)	(F,N,N)
(F,F,F)_(F,P,P)	1.000	1.000	1.000	W2_W1	0.886	0.757	0.002**
(F,P,P)_(F,N,N)	0.317	0.013*	0.007**	W3_W1	0.868	0.810	0.036*
(F,F,F)_(F,N,N)	0.471	<0.001***	0.002*	W3_W2	0.771	0.615	0.699
TMIG-IC							
Status	W1	W2	W3	Time	(F,F,F)	(F,P,P)	(F,N,N)
(F,F,F)_(F,P,P)	0.494	1.000	1.000	W2_W1	0.534	0.510	<0.001***
(F,P,P)_(F,N,N)	0.02**	0.002**	<0.001***	W3_W1	0.004**	0.559	<0.001***
(F,F,F)_(F,N,N)	0.105	<0.001***	<0.001***	W3_W2	<0.001***	0.870	0.012
(F,N,N)(P,N,N)							
Self-rated health							
Status	W1	W2	W3	Time	(F,N,N)	(P,N,N)	
(F,N,N)_(P,N,N)	1.000	1.000	0.149	W2_W1	0.517	0.663	
				W3_W1	0.573	0.023*	
				W3_W2	0.196	0.002**	
GDS							
Status	W1	W2	W3	Time	(F,N,N)	(P,N,N)	
(F,N,N)_(P,N,N)	1.000	0.216	1.000	W2_W1	0.013*	0.051	
				W3_W1	0.043*	0.002**	
				W3_W2	0.701	0.159	
TMIG-IC							
Status	W1	W2	W3	Time	(F,N,N)	(P,N,N)	
(F,N,N)_(P,N,N)	0.281	0.003*	<0.001***	W2_W1	<0.001***	0.719	
				W3_W1	<0.001***	0.004**	
				W3_W2	0.019*	0.002**	

F: Full-time worker, P: Part-time worker, N: Non-worker.

* p<0.05

**p<0.01

***p<0.001 was applied by t-tests with Bonferroni Corrections.

## Discussion

According to the cross-sectional analysis, working over 65 years old relates significantly to the high scores of self-rated health, mental health (GDS15), and HLFC (TMIG-IC). Part-time workers attained almost equal scores as full-time workers. According to the longitudinal analysis of comparison shown at (Fig 2), we found a causal relationship that retirement has a bad effect on both mental health and HLFC, but it doesn’t significantly have an effect on self-rated health. On the other hand, the people who changed their jobs from full-time to part-time were not affected, which is the same as the people who continued working full-time. In the comparison of the two groups in Fig 3, the effect of quitting from their part-time job on mental health was slower than that of full-time job but reached the same level in a long-term. On the contrary, the effect on HLFC started 2 years after Wave 1.

The fact that retirement leads to the deterioration of one’s health, after age adjustment means that working has a good effect to maintain one’s health. Consequently, this study supports the activity theory of working people over 65 years old.

We have considered five reasons for it. First, the characteristics of the jobs they engage are different. For instance, the jobs for those 65 years older tend to be generally very easy. They don’t feel nervous doing these jobs. That may be why they don’t feel relax when quitting their jobs.

Secondly, the attitudes the jobs require are also different; the former jobs are their lifelong jobs to earn livings but the latter usually means only an addition. They are not required to work at responsible positions with high pressure but as individuals outside the seniority system such as guard, garbage collector, cook, or caretaker of an apartment [22]. That would be why part-time job also has almost the same effects.

Thirdly, retirement may cause the secession from the effects of social participation to health. They may have lost the connection to society by retirement which can only be maintained by engaging to work. This study shows that the effects of retirement both on mental health and HLFC occur at different speed. The long-term effects on physical and functional capabilities appear slower than mental aspects. That may be because isolation proceeds slowly and harms HLFC correspondingly after losing the connection with the society.

Fourthly, it may by a reverse causation that poor health causes retirement. By the multiple comparison of each groups by each waves (Table 2), we can find one significant value between (F,P,P) and (F,N,N) at Wave1. Whether older people choose part-time job or retirement at quitting from full-time job may be affected by the health status and if they choose retirement, the health status becomes worse. Other reverse causations are not seen statistically.

Fifthly, there may be some intermediate factors. For example, the decrease of income due to retirement may be the reason of health deterioration. Actually, there is a significant difference of the annual couple income between (F,F,F) and (F,P,P) or (F,N,N)at Wave 2 (p<0.001, p = 0.017), but the correlations between the annual income and GDS or HLFC at Wave 2 are not statistically significant. Therefore, the possibility of income decreases as the intermediate factor is limited.

Apart from that, we have clarified the effect of part-time job. Although there are many older job-seekers for full-time jobs [22], it seems to be enough to maintain their health by doing part-time jobs and a good transition when quitting from full-time job to avoid rapid deterioration of health in those over 65 years old.

This study has various limitations. This research was conducted only 3 times in 4 years. We must examine whether the effects are only temporal or permanent by conducting longer researches. The interval is also 2 years, so the definition of short-term and long-term is by the convenience of statistical operations. Also, the variety of occupations was not considered. Self-employment, which is contained statistically more in the subjects of this research, or farming, which is more in rural areas, may be different from other occupations. The sample sizes of the ANCOVAs are relatively small even though they are statistically significant. Although this analysis was based on a large quantity of the samples, it is expected to be supplemented to raise the precision of the results by the additional research, for example, by the qualitative studies.

In Japan, the policy called “community care system” is promoting for 2025 as the solution of our unprecedented ageing society. Engagement in the work of older people is expected to be a part of it as one of the variety programs to prompt older people to social participations. Working is expected to be higher and, thus, be regarded as the most productive activity than volunteer, hobby, or other programs. So, the fact that working is desirable to maintain one’s health as this study clarified should be known widely.

Nevertheless, at the same time, retirement over 65 years old leads to deterioration of one’s health. To maintain their health even after retirement, we are expected to support them a seamless shift to the next program of social participation [29]. Thereby, the studies about the comparison or the relation between working and other programs are expected after this.

## Supporting Information

S1 File Table A Descriptive statistics before adjustments by covariates. F: Full-time worker, P: Part-time worker, N: Non-worker, GDS: the short form of Geriatricl Depression Scale, TMIG-IC: TMIG Index of Competence to measure Higher-Level Functional Capacity. Table B Effect sizes of two analysis by repeated measures ANCOVA: (F,F,F)(F,P,P)(F,N,N) and (F,N,N)(P,N,N). DV: dependent variable, SS: Type III Sum of Squares, df: degrees of freedom, MS: Mean Squares, F: F value, p: tested by F test; * p<0.05, **p<0.01, ***p<0.001, pη2: partial η squared. 5 covariates were used of Sex, age, years of schooling, annual couple income, occupation (self-employed or not). F: Full-time worker, P: Part-time worker, N: Non-worker, GDS: the short form of Geriatric Depression Scale, TMIG-IC: TMIG Index of Competence to measure Higher Level Functional Capacity. (DOCX)
