# Supplementary material for: Effects of the Change in Working Status on the Health of Older People in Japan
Source: PLoS One. 2015 Dec 3;10(12):e0144069. doi: 10.1371/journal.pone.0144069 (PMC4669179; doi:10.1371/journal.pone.0144069)
Supplement: S1 File — (DOCX) [file pone.0144069.s001.docx]

Table A Descriptive statistics before adjustments by covariates

|  |  | Self-rated health | | | | | | GDS | | | | | | TMIG-IC | | | | | |
| --- | --- | --- | --- | --- | --- | --- | --- | --- | --- | --- | --- | --- | --- | --- | --- | --- | --- | --- | --- |
|  |  | W1 | | W2 | | W3 | | W1 | | W2 | | W3 | | W1 | | W2 | | W3 | |
|  | N | M | SD | M | SD | M | SD | M | SD | M | SD | M | SD | M | SD | M | SD | M | SD |
| (F,F,F) | 55 | 0.91 | 0.30 | 0.91 | 0.29 | 0.89 | 0.31 | 3.52 | 2.11 | 3.56 | 2.13 | 3.46 | 2.09 | 12.30 | 1.11 | 12.40 | 1.00 | 11.63 | 1.67 |
| (F,P,P) | 13 | 0.93 | 0.27 | 1.00 | 0.00 | 0.93 | 0.27 | 3.14 | 1.56 | 3.38 | 1.56 | 3.21 | 1.89 | 12.79 | 0.58 | 12.57 | 0.76 | 12.50 | 0.85 |
| (F,N,N) | 20 | 0.81 | 0.40 | 0.71 | 0.46 | 0.74 | 0.45 | 4.63 | 3.10 | 5.96 | 3.78 | 5.74 | 3.85 | 11.50 | 1.91 | 10.46 | 3.36 | 9.48 | 3.37 |
| (P,N,N) | 41 | 0.78 | 0.42 | 0.82 | 0.39 | 0.68 | 0.47 | 4.41 | 3.12 | 5.02 | 2.91 | 5.44 | 3.25 | 11.93 | 1.71 | 11.92 | 1.76 | 11.09 | 2.45 |
| Average | 1768 | 0.76 | 0.43 | 0.79 | 0.40 | 0.77 | 0.42 | 4.81 | 3.12 | 4.76 | 3.07 | 4.77 | 3.04 | 11.04 | 2.78 | 11.30 | 2.60 | 10.82 | 2.78 |

F: Full-time worker, P: Part-time worker, N: Non-worker, GDS: the short form of Geriatricl Depression Scale, TMIG-IC: TMIG Index of Competence to measure Higher Level Functional Capacity

Table B Effect sizes of two analysis by repeated measures ANCOVA: (F,F,F)(F,P,P)(F,N,N) and (F,N,N)(P,N,N)

| Source | *DV* | *SS* | *df* | *MS* | *F* | *p* | *pη2* | *SS* | *df* | *MS* | *F* | *p* | *pη2* |
| --- | --- | --- | --- | --- | --- | --- | --- | --- | --- | --- | --- | --- | --- |
|  | | Between Subjects (F,F,F)(F,P,P)(F,N,N) | | | | | | Between Subjects (F,N,N)(P,N,N) | | | | | |
| Status | Self-rated health | 0.420 | 2 | 0.210 | 1.077 | 0.346 | 0.031 | 0.230 | 1 | 0.230 | 0.650 | 0.424 | 0.014 |
|  | GDS | 186.867 | 2 | 93.434 | 6.802 | 0.002** | 0.154 | 28.897 | 1 | 28.897 | 1.161 | 0.286 | 0.021 |
|  | TMIG-IC | 168.267 | 2 | 84.134 | 11.281 | <0.001*** | 0.220 | 111.133 | 1 | 111.133 | 9.131 | 0.004** | 0.147 |
| Error | Self-rated health | 13.262 | 68 | 0.195 |  |  |  | 16.615 | 47 | 0.354 |  |  |  |
|  | GDS | 1030.152 | 75 | 13.735 |  |  |  | 1319.566 | 53 | 24.897 |  |  |  |
|  | TMIG-IC | 596.627 | 80 | 7.458 |  |  |  | 645.049 | 53 | 12.171 |  |  |  |
|  | | Within Subjects (F,F,F)(F,P,P)(F,N,N) | | | | | | Within Subjects (F,N,N)(P,N,N) | | | | | |
| Time | Self-rated health | 0.160 | 1.661 | 0.096 | 1.365 | 0.258 | 0.020 | 0.094 | 2 | 0.047 | 0.547 | 0.581 | 0.012 |
|  | GDS | 4.892 | 1.825 | 2.681 | 1.081 | 0.337 | 0.014 | 14.430 | 2 | 7.215 | 1.997 | 0.141 | 0.036 |
|  | TMIG-IC | 0.579 | 1.762 | 0.329 | 0.243 | 0.757 | 0.003 | 2.385 | 2 | 1.192 | 0.53 | 0.590 | 0.010 |
| Time*Status | Self-rated health | 0.126 | 3.323 | 0.038 | 0.541 | 0.673 | 0.016 | 0.687 | 2 | 0.344 | 4.009 | 0.021* | 0.079 |
|  | GDS | 25.329 | 3.65 | 6.940 | 2.799 | 0.033* | 0.069 | 3.009 | 2 | 1.505 | 0.417 | 0.660 | 0.008 |
|  | TMIG-IC | 24.051 | 3.524 | 6.826 | 5.044 | 0.001** | 0.112 | 12.796 | 2 | 6.398 | 2.844 | 0.063 | 0.051 |
| Error(Time) | Self-rated health | 7.952 | 112.98 | 0.070 |  |  |  | 8.058 | 94 | 0.086 |  |  |  |
|  | GDS | 339.408 | 136.873 | 2.480 |  |  |  | 382.944 | 106 | 3.613 |  |  |  |
|  | TMIG-IC | 190.718 | 140.94 | 1.353 |  |  |  | 238.478 | 106 | 2.25 |  |  |  |
| Total | Self-rated health | 21.920 | 187.964 |  |  |  |  | 25.684 | 146 |  |  |  |  |
|  | GDS | 1586.648 | 219.348 |  |  |  |  | 1748.846 | 164 |  |  |  |  |
|  | TMIG-IC | 980.242 | 228.226 |  |  |  |  | 1009.841 | 164 |  |  |  |  |

DV: dependent variable , SS: Type III Sum of Squares , df: degrees of freedom, MS: Mean Squares , F: F value, p: tested by F test; * p<0.05, **p<0.01, ***p<0.001 , pη2: partial η squared. 5 covariates were used of Sex, age, years of schooling, annual couple income, occupation (self-employed or not). F: Full-time worker, P: Part-time worker, N: Non-worker, GDS: the short form of Geriatric Depression Scale, TMIG-IC: TMIG Index of Competence to measure Higher Level Functional Capacity.
